# Improving the transition from medical student to junior doctor: a one-month course in the final year of medical school

**DOI:** 10.15694/mep.2017.000026

**Published:** 2017-02-08

**Authors:** Eve Boakes, Nikita Shah

**Affiliations:** 1 London North West Healthcare Trust

**Keywords:** Transition, Preparation course, Junior doctor, Improving confidence

## Abstract

This article was migrated. The article was marked as recommended.

Concern exists that the transition from student to doctor is abrupt and stressful, with new graduates lacking both clinical skills and confidence. This study explores the effect a preparation programme can have on the confidence and skills of final year medical students (FYMSs), prior to commencing their first clinical post. Foundation year one (FY1) doctors were surveyed on challenges they faced when commencing clinical work. Findings were used to design a practical, four-week, eight lecture course, aimed at preparing final year medical students for work. Questionnaires and focus groups were used to establish pre- and post-course concerns.

Amongst FY1 doctors (n=105) the predominant concern was the diagnosis and management of unwell patients (66.7%). Medical students expressed similar fears (80.85%). On average each session significantly improved confidence levels by 25.3% (95% CI: 23.27-27.12%, p<0.01). Sessions on prescribing and palliative care showed greatest confidence improvement (31.1% and 29.4% respectively).

This programme supports the transition from medical student to practising doctor, and was found to be useful and effective at building student confidence through practical advice from current FY1 doctors to the next generation of junior doctors. It remains unclear as to when (within the year) this course would be most beneficially placed.

## Introduction

Concern exists that the transition from student to doctor is abrupt and stressful, with new graduates lacking both clinical skills and confidence ( Morrow *et al.* 2012; Berridge *et al.* 2007). It has also been shown that incoming doctors often lack particular skills in the diagnosis and management of unwell patients in the acute care ( Tallentire *et al.* 2012; Tallentire *et al.* 2011). Dealing with unwell patients is something junior doctors will face immediately upon commencing clinical work. One systematic review suggested that recent changes in UK undergraduate training, while improving preparedness in some areas, may have neglected acute care ( Tallentire *et al.* 2012). While not a good surrogate for actual preparedness, perceived preparedness is important in influencing the behaviour of new graduates and therefore warrants further consideration ( Tallentire *et al.* 2012). Previous work has shown that induction courses immediately prior to FY1 help to prepare students for clinical work ( Berridge *et al.* 2007). However this is variable and it draws the question that if students were better prepared during medical school, would they need this last minute induction course?

Evaluation is an integral part of medical education. Despite a wide use of various evaluation tools, little is known about student perceptions regarding the purpose and desired consequences of evaluation. One study found that questionnaires and focus groups have been shown to be effective ways of assessing student confidence and feedback ( Schiekirka et al. 2012). This study uses questionnaires and study groups to assess the impact of a FY1 preparation course on the confidence and skills of final year medical students, prior to commencing their first clinical post.

## Materials and Methods

**Figure 1.**
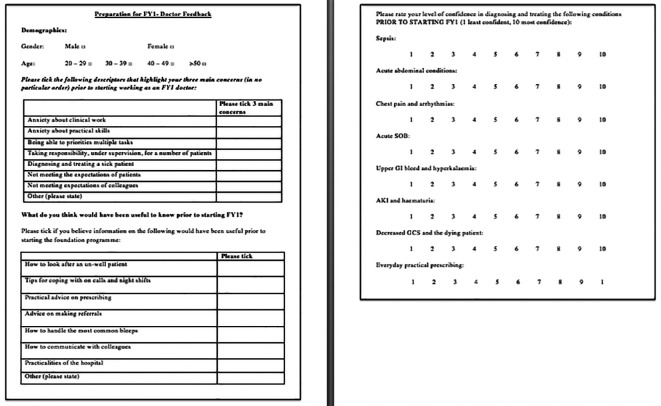
Questionnaire used to analyse concerns of FY1 doctors prior to starting clinical work (a similar questionnaire was used to analyse concerns of FYMSs).

FY1 doctors were surveyed on challenges they faced when commencing clinical work, their initial confidence in managing unwell patients and what they thought would have been useful to know prior to starting FY1. The questionnaire used can be seen in Figure 1. Findings were used to design a practical, four-week, eight-session course, aimed at preparing FYMS for clinical work. Each session included a 20-minute didactic lecture and then students working through clinical cases/scenarios as a group. All sessions were taught by current FY1 doctors and incorporated; how to approach on-call shifts, answering the bleep, prioritising tasks, presenting patients and communicating with patients and colleagues. These skills were developed session-by-session using topics that highlighted the practicalities of diagnosing and managing unwell patients.

The topics covered in each session were:

1. Sepsis (approach to mild and severe cases (septic shock))
2. Acute abdomen (cases including appendicitis, pancreatitis, peptic ulcers and diverticulitis)
3. Shortness of breath (SOB) (cases including asthma, pulmonary oedema and pulmonary embolism)
4. Chest pain and arrhythmias (cases including Acute Coronary Syndrome (ACS) and atrial fibrillation)
5. Acute Kidney Injury (AKI) and haematuria (including approach to medications in AKI)
6. Hyperkalaemia and upper GI bleed (UGIB)
7. Low Glasgow Coma Scale (GCS) and the dying patient (including prescribing in palliative care)
8. Practical prescribing; including analgesia, antibiotics, fluids, insulin and warfarin.

**Figure 2.**
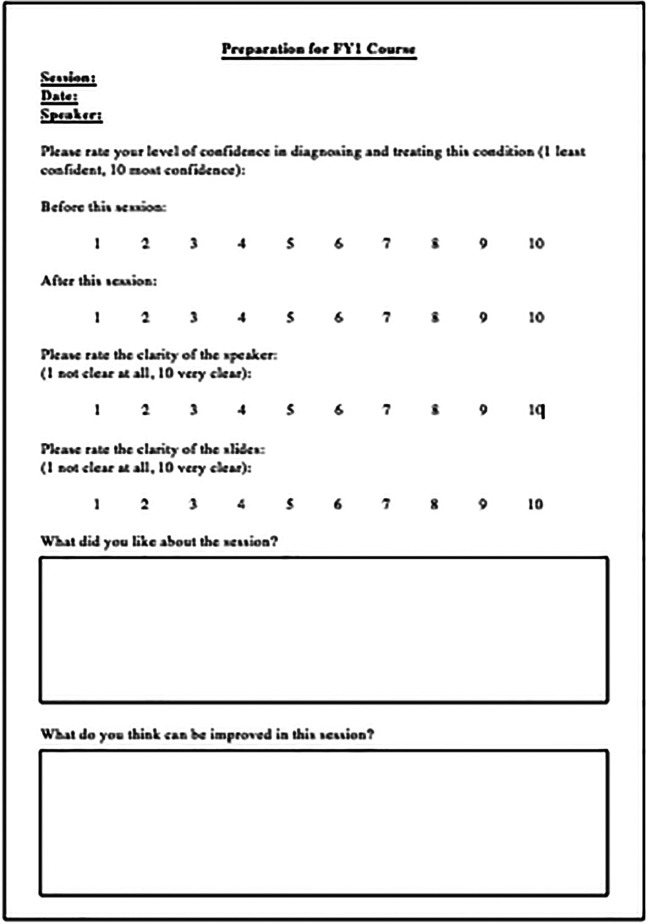
Questionnaire used to analyse FYMSs feedback from each session.

Questionnaires and focus groups were used to establish concerns pre-course (the questionnaire used was similar to the one shown in Figure 1), and how confidence improved afterwards (scale 0 (least confident)-10 (most confident)). The post course questionnaire used can be seen in Figure 2. Ten focus groups were carried out; one at the beginning and one at the end of each course. Students had the opportunity to discuss their concerns about clinical work and how they thought the course had improved their confidence. We also discussed their specific likes and dislikes about the course. Statistical analyses were performed in Excel (Microsoft Office, Washington, USA) and R-Studio (Boston, USA) and 95% confidence intervals were calculated. A paired t-test was performed using the null hypothesis there is no difference in mean pre and post course marks. Significance was set at p-values less than 0.01.

## Results

### Concerns prior to starting clinical work

**Figure 3.**
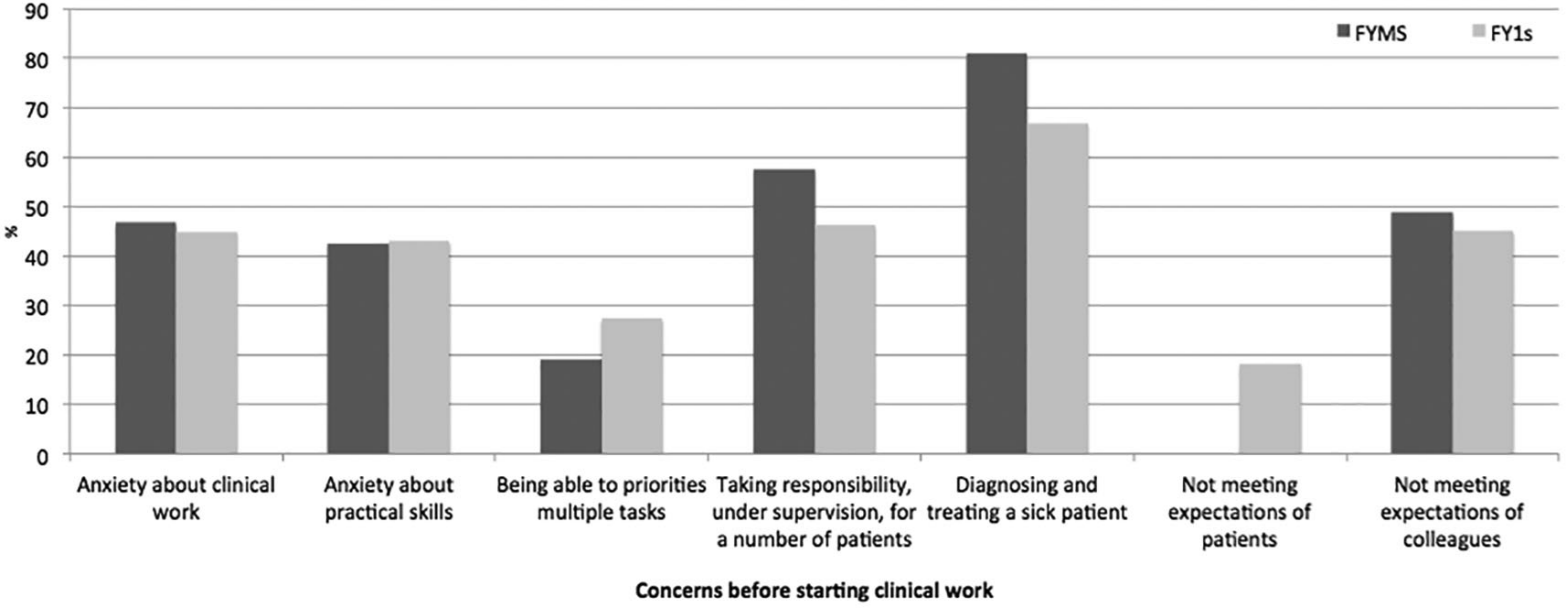
Retrospective concerns of FY1 doctors and prospective concerns of FYMSs prior to starting clinical work.

The opinions from 105 FY1 doctors from 25 different medical schools (m=56, f=49) and 47 Imperial College FYMSs (m=20, f=27) were analysed. Predominant concerns were; the diagnosis and management of unwell patients (FY1: 66.7%, FYMS: 80.9%), taking responsibility for a number of patients (FY1: 46.3%, FYMS: 57.4%) and not meeting expectations of colleagues (FY1: 45.1%, FYMS: 48.9%) ( Figure 3).

**Figure 4.**
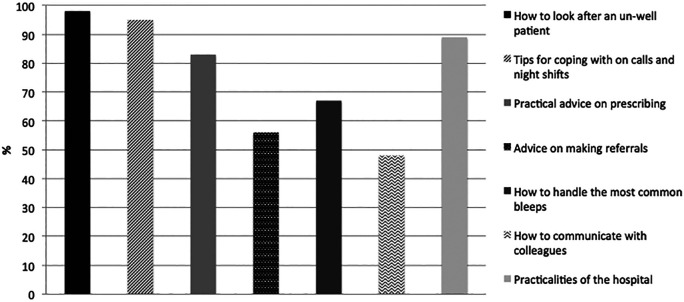
Information current FY1’s thought would be useful for those starting clinical work (n=105).

Current FY1s were also asked what they thought would be helpful to know prior to starting clinical work and what they thought their own medical student education lacked ( Figure 4).

This information ( Figure 3 and 4) was collated and used to design a teaching course for FYMSs to try and help ease some of these fears and provide useful advice which may not be readily accessible from their traditional medical school course.

### Impact of teaching programme

**Figure 5.**
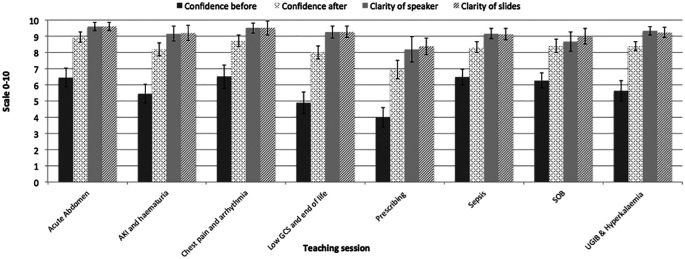
How FYMSs rated their confidence before and after sessions and the quality of the teaching (Scale 0-10).

The course was delivered over the final year of study. It consisted of 8 sessions over a one-month period on a rolling programme that corresponded with clinical rotations at Ealing Hospital, London, UK. Forty sessions were delivered in total. Ten focus groups were carried out (one before and one at the end of each course). A pre-course questionnaire was completed and a separate questionnaire was used to gain feedback for each individual session (total questionnaires collected = 154). The level of confidence before and after each session can be found in Figure 5. The average confidence level prior to teaching was 5.7 (range 4.5-6.6/10). Students felt least confident in prescribing (4/10). Students felt most confident in diagnosing and managing chest pain and arrhythmias (6.5/10). On average each session improved the level of confidence by 25.3% (CI=23.3-27.1%) (p=<0.01). Sessions on low GCS and the dying patient and prescribing showed greatest improvement in confidence (31.1% (p=<0.01)) and 29.4% (p=<0.01) respectively. Using paired-tests each individual session showed a significant increase in confidence (p=<0.01). Students felt that the clarity of speaker and slides were excellent (clarity of speaker: 8.2-9.6/10, clarity of slides: 8.3-9.6/10) ( Figure 5).

Focus groups allowed us to discuss the pros and cons of the course; the most common themes were:

• Students liked clinical scenarios and interactive nature
• Students liked that it was taught by current FY1 doctors making the content more tailored and teachers more approachable
• Students like the didactic section to be short and have clear management plans that they could follow in practice
• The majority of students would have liked a hand-out to accompany the session.

## Discussion

There are clear shortcomings in medical student education as the current system fails to empower future doctors to diagnose and manage the un-well patient ( Morrow *et al.* 2012). By encouraging feedback from current FY1 doctors and FYMSs it was possible to develop a course which was directed towards the students particular concerns. Focusing on the management of acutely unwell patients boosted student confidence in this area (a known weakness of newly qualified doctors ( Tallentire *et al.* 2011; Tallentire *et al.* 2012)). Providing a preparation course in the final year of study increased confidence and the programme’s content directly mitigated some fears. Being an FY1 led course made students feel that the content was more tailored and teachers more approachable, an important factor when delivering content which aims to improve student confidence. A course like this provides a valuable adjunct to traditional medical school curriculum, which is important to deliver prior to starting FY1. Incorporating it into the timetable for all students in the final year of study may help to better prepare students for their first clinical jobs. It remains unclear whether it may be more beneficial to incorporate these sessions into the FY1 shadowing period so that information is more fresh in their minds and specific to each individual’s own hospital upon commencement of clinical work. However, as this study has established FYMSs have real concerns and worries about starting clinical work it may be prudent to mitigate these fears earlier. A combination of this course during the final year of study with a refresher during the induction period may provide the most beneficial student experience.

By directly establishing the concerns of FY1 doctors and FYMS about starting clinical work, it was possible to tailor this FY1 led course accordingly. This programme supports the transition from medical student to practising doctor, and was found to be useful and effective at building student confidence through practical advice from current FY1 doctors to the next generation of junior doctors.

## Take Home Messages

• The transition from medical student to junior doctor is a difficult one.
• By analysing worries of students and junior doctors prior to starting clinical work a course was designed to target and plicate these fears.
• This course was delivered by FY1 doctors to students in their final year of study and questionnaires and focus groups were used to assess feedback.
• The course improved the confidence of students by an average of 25.3% (p<0.01).
• This study shows that a valuable, targeted and interactive course can greatly improve the confidence and transition of medical students to junior doctors.

## Notes On Contributors

Dr Eve Boakes and Dr Nikita Shah are currently working in hospitals in North West Thames (UK) completing their second year of the Foundation Programme. They both enjoy teaching and research in addition to clinical practice. They are particularly interested in improving the transition from medical student to junior doctor.

## References

[1] Morrow G. Preparedness for practice: the perceptions of medical graduates and clinical teams. Med. Teach. 34,123–35(2012). 10.3109/0142159X.2012.643260 22288990

[2] Berridge E.-J. Freeth D. Sharpe J. & Roberts C. M. Bridging the gap: supporting the transition from medical student to practising doctor - a two-week preparation programme after graduation. Med. Teach. 29,119–127(2007). 10.1080/01421590701310897 17701621

[3] Tallentire V. R. Smith S. E. Wylde K. & Cameron H. S. Are medical graduates ready to face the challenges of Foundation training? Postgrad. Med. J. 87,590–5(2011). 10.1136/pgmj.2010.115659 21690255

[4] Tallentire V. R. Smith S. E. Skinner J. & Cameron H. S. The preparedness of UK graduates in acute care: a systematic literature review. Postgrad. Med. J. 88,365–71(2012). 10.1136/postgradmedj-2011-130232 22167809

[5] Schiekirka S. Student perceptions of evaluation in undergraduate medical education: A qualitative study from one medical school. BMC Med. Educ. 12,45(2012). 10.1186/1472-6920-12-45 22726271 PMC3408338

