# Supplementary material for: Improving the transition from medical student to junior doctor: a one-month course in the final year of medical school
Source: MedEdPublish (2016). 2017 Feb 8;6:26. [Version 1] doi: 10.15694/mep.2017.000026 (PMC10885232; doi:10.15694/mep.2017.000026)
Supplement: Peer review history

# Reviewer report: Reviewer response for version 1

**DOI:** 10.21956/mep.19410.r28484

**Published:** 2017-02-17

**Authors:** Suzanne Lady

**Affiliations:** 1 University of Western States

This review has been migrated. The reviewer awarded 4 stars out of 5 Student to doctor transitions are always difficult and any laudable solution to help this transition is ultimately helpful. I think the authors did a great job of recognizing the value of metacognition in the student and surveying them directly about what they felt they needed the most to make this transition. The thing that surprises me the most is that an 8 session lecture series could improve the confidence in these students significantly. These same students have likely had many lecture style courses in their medical academic careers, why was this one more impactful? Maybe because the course was designed with direct student input or maybe because their transition was on the near horizon which created a more hyper focused learning environment. Knowing the circumstances around events can be helpful for the future success of medical course design and function. It almost seems reasonable instead of waiting until students make this transition to start earlier in programs giving students opportunities to practice acute care skills so when the time comes the confidence can be built upon, not created in a month. Because the cognitive lecture reviews were so helpful, I can’t help but wonder if combining this with practical scenarios would have improved their confidence levels even further. The use of standardized patient encounters into each of the acute care topics in that month period leading up to their transition may have moved that 25% even higher. Good paper for those interested in helping ease the transition of student to doctor.

Reviewer Expertise:

NA

No decision status is available

# Reviewer report: Reviewer response for version 1

**DOI:** 10.21956/mep.19410.r28483

**Published:** 2017-02-09

**Authors:** Subha Ramani

**Affiliations:** 1 Harvard Medical School, Brigham and Women's Hospital

This review has been migrated. The reviewer awarded 4 stars out of 5 All transitions are challenging and the reasons for the anxiety around this transition from medical student to junior doctor are appropriate. While I agree that medical students can be given increased patient care responsibilities / ownership, junior and senior doctors are still accountable for patient care in the eyes of society and the law. Thus, the transition stages can be fraught with anxiety. I enjoyed reading this paper and commend the authors for researching and reporting this topic. The abstract sets the tone for the rest of the paper. In the second paragraph of the intro, the abrupt jump to evaluation without a lead in feels a little out of place.I like the study methods section detailing the exploration of junior doctor concerns and designing the course based on these concerns. The one thing I would recommend is authors should justify data collection methods- questionnaires and focus group discussions, particularly the focus groups. Did they expect that group interactions would yield more in-depth discussions? Justification of choice of data collection methods is important especially when collecting qualitative data.The topics chosen seem very appropriate and the combination of didactics and case discussions would be very useful.The junior doctor perceptions of challenges and concerns are valuable. Program evaluation exploring self-reported confidence pre and post course implementation is a good start.I hope the authors plan to continue their explorations on this topic. Specifically, the following areas would be important:1. the course- beyond didactics and case discussions, simulations would be valuable for students to practice caring for acutely ill patients and teamwork.2. program evaluation beyond self reported confidence and satisfaction. Higher levels of the Kirkpatrick pyramid should be considered- improvement in knowledge, change in behavior etc3. use of focus groups should truly attempt to extract rich narrative data that would be difficult to obtain otherwise. Focus groups before and after each topic probably are redundant.This paper can provide many lessons for other educators working in this area. But I would like to emphasize that this is a start, good start, but the curriculum should continue to grow and evaluation of the program should target higher level outcomes.

Reviewer Expertise:

NA

No decision status is available

# Reviewer report: Reviewer response for version 1

**DOI:** 10.21956/mep.19410.r28486

**Published:** 2017-02-08

**Authors:** Richard Hays

**Affiliations:** 1 James Cook University

This review has been migrated. The reviewer awarded 3 stars out of 5 Thanks from this 'view from the trenches'. The perspectives on work readiness from recent graduates are interesting and valuable, and the program described appears to be sound. However, I cannot help wondering why there needs to be so much angst at this transition point. A primary medical qualification is about preparation for a long career potentially in many diverse specialties. It used to be that the final medical school year was an immersion in clinical practice and the first postgraduate year was the 'finishing school' where graduates developed skills and confidence It seems that now on day 1 graduates are expected, or at least feel that they are expected, to be relatively expert in managing rather complex acute or emergency hospital-based scenarios. One learns best when there is some responsibility for outcomes, and yet responsibility appears to have been largely removed from medical students and increased for PGY1 doctors. 'Bridging' courses like this then become popular and perhaps necessary, but perhaps a re-design of final year and PGY1 would achieve a smoother transition?

Reviewer Expertise:

NA

No decision status is available

# Reviewer report: Reviewer response for version 1

**DOI:** 10.21956/mep.19410.r28485

**Published:** 2017-02-08

**Authors:** Trevor Gibbs

**Affiliations:** 1 AMEE

This review has been migrated. The reviewer awarded 4 stars out of 5 I enjoyed reading this paper, which, when judged by the number of similar papers on the same subject, reflects its importance.It was also good to see that a poster presented at the AMEE 2016 annual conference in Barcelona as a poster had now been converted into a paper - well doneThe paper, as described above, covered that important transition phase from student to doctor, from final year student to Year 1 Foundation Year Doctor (FY 1) in this case. It was very relevant that the authors structured their course around a needs assessment ( actually more of a worry assessment !) developed from existing FY1 doctors. Although there are many papers now, some of which are not in the reference list, that describe similar courses, I felt that this paper was very clearly written and structured and would be of great help to those considering similar courses.I do think that it raises other issues, such as why if our curricula are that well structured, do we still have this worry from young doctors?I would like to see a follow-on from this paper exploring the impact that the course had on the students once they had graduated- improving confidence is good, translating that improved confidence into confident action could be even better

Reviewer Expertise:

NA

No decision status is available
